# Impact of health disparities on national breast cancer screening participation rates in South Korea

**DOI:** 10.1038/s41598-023-40164-y

**Published:** 2023-08-14

**Authors:** Fatima Nari, Juwon Park, Nayeon Kim, Dong Jin Kim, Jae Kwan Jun, Kui Son Choi, Mina Suh

**Affiliations:** 1 National Cancer Control Institute, National Cancer Center, 323-Ilsan-Ro, Goyang, 10408 Republic of Korea; 2 Center for Health Policy Research, Korea Institute for Health and Social Affairs, Sejong City, 30147 Republic of Korea; 3 Graduate School of Cancer Science and Policy, National Cancer Center, Goyang, 10408 Republic of Korea

**Keywords:** Breast cancer, Cancer screening, Health services, Public health

## Abstract

Socioeconomic barriers to cancer screening exist at a regional level. The deprivation index is used to estimate socioeconomic gradients and health disparities across different geographical regions. We aimed to examine the impact of deprivation on breast cancer screening participation rates among South Korean women. Municipal breast cancer screening participation rates in women were extracted from the National Cancer Screening Information System and linked to the Korean version of the deprivation index constructed by the Korea Institute for Health and Social Affairs. A generalised linear mixed model was employed to investigate the association between the deprivation index and age-standardised breast cancer screening participation rates in 2005, 2012, and 2018. Participation rates increased gradually across all age groups from 2005 to 2018. Participants in their 60 s consistently had one of the highest participation rates (2005: 30.37%, 2012: 61.57%, 2018: 65.88%). In 2005, the most deprived quintile had a higher estimate of breast cancer screening participation than the least deprived quintile (2nd quintile; estimate: 1.044, p = 0.242, 3rd quintile; estimate: 1.153, p = 0.192, 4th quintile; estimate: 3.517, p = 0.001, 5th quintile; estimate: 6.913, p =  < 0.0001). In 2012, the participation rate also increased as the level of deprivation increased. There were no statistically meaningful results in 2018. Regions with high deprivation have a higher participation rate in breast cancer screening. The role of health disparities in determining cancer outcomes among women in Korea requires further examination.

## Introduction

Breast cancer remains one of the most common cancers affecting women globally and in Korea^1,2^. Although mortality due to breast cancer has gradually declined, the increasing incidence of breast cancer among women has become a growing concern, as it may pose a substantial burden on the population and the healthcare system^3,4^.

To tackle this issue, the National Cancer Center in Korea first introduced the National Cancer Screening Program (NCSP) in 2002, which provides population-based organised cancer screening for five cancer types, including breast cancer^5^. The NCSP guidelines state that breast cancer screening is provided to women in Korea over the age of 40 years, and that screening is performed biennially, using mammography as the screening modality^6^. Furthermore, this program aims to improve cancer prognosis and survival outcomes through early detection and charges little to no money for its services to ensure that the entire population is able to undergo cancer screening^1,7^.

Despite this, existing socioeconomic barriers may limit the use of cancer screening services and result in non-compliance with screening guidelines^3,8,9^. Examples of such barriers include access to health care resources, income-related and education-related disparities^10^. A prior study in Korea examined the influence of various socioeconomic factors on having received a mammogram and concluded that low socioeconomic status is associated with disparities in breast cancer screening^11^.

Disparities in access to healthcare services have been reported in urban and rural settings^8^. One measure commonly used by researchers in public health research to quantify socioeconomic disparities in geographical areas is deprivation^12,13^. The concept of deprivation first originated from the Townsend definition, which refers to “a state of observable and demonstratable disadvantage relative to the local community or the wider society or nation to which an individual, family, or group belongs”^14^. Deprivation is an approach that reflects socioeconomic position by taking into account actual living conditions, which is closely linked to health status^15^. An area with high level of deprivation is described as a geographical unit in which a majority of inhabitants are characterised with low socioeconomic status, such as unemployment, low educational level, low income…etc^16^. Thus, the level of deprivation in different regions is calculated based on the assumption that individuals in smaller area units are likely to have similar social environments and socioeconomic conditions^17,18^. Commonly used indices developed to assess area level socioeconomic deprivation include the Townsend Index, Carstairs Index and Jarman Index^18^.

It is essential to address how health disparities within specific geographical units such as municipal level areas influence health outcomes in the population. Therefore, this study’s aim was to investigate how deprivation influences breast cancer screening participation rates in South Korean women, using national cancer screening data.

## Materials and methods

Our study’s target population consisted of women aged ≥ 40 years invited to participate in the national breast cancer screening in all municipal level regions in South Korea. These invited participants comprised National Health Insurance Service beneficiaries within the lower 50% income bracket and Medical Aid recipients. The National Cancer Center started releasing annual breast cancer screening statistics from 2005, where screenings conducted between January 1st and December 31st of the corresponding year are recorded^19^. Breast cancer screening participation rates were extracted using data from the National Cancer Screening Information System, and denoted as the percentage of women who underwent screening from the total invited population. Age-standardized participation rates were obtained by incorporating the population size of the year that the screening was conducted in. Participants screened in 2005, 2012, and 2018 were included to observe differences in influence of deprivation on screening participations rates at various points in the NCSP timeline i.e. early-, middle- and advanced- stages since the NCSP launch, using latest available data. 2002, 2003 and 2004 breast cancer screening data were excluded due to irregularity of data. Furthermore, the 2018 Korean deprivation index provided the most recent deprivation data for our study. The number of women invited to participate in breast cancer screening in 2005, 2012 and 2018 were 3,015,882, 3,577,065, and 4,042,332 individuals respectively. Trend analysis results on national breast cancer screening rates were provided elsewhere^5^. This study was conducted in accordance with the Declaration of Helsinki, and approved by the National Cancer Center Institutional Review Board of Korea (approval number: NCC2021-0264, approval date: 2022.08.24).

The Korean version of the deprivation index, developed by the Korea Institute for Health and Social Affairs, is a continuous measure for quantifying regional health disparities by considering a total of nine different socioeconomic determinants including living alone, being part of the older adult population, not owning a car, not residing in an apartment, having an underdeveloped living environment, being part of the divorced or widowed population, unemployment, having low educational attainment, and low social class^20^. Increasing deprivation index values indicated higher deprivation levels (with values ranging − 20.3312387 to 15.1452312). Korea comprises 250 municipal level units (otherwise known as Sigungu), and deprivation and screening participation data were provided for all municipal level areas.

We divided the deprivation index into quintiles (1 = least deprived to 5 = most deprived), where 20% of the municipal level units with the lowest deprivation scores fell into the 1st quintile and 20% of municipal level units with the highest deprivation scores were categorised in quintile 5. We then investigated its effect on breast cancer screening participation rates using a generalised linear mixed model (GLMM). The GLMM is a flexible parametric model that is an extension of the generalised linear model with added random effects, that allows for analysis of data that follow a non-normal distribution^21^. We included a variable that depicts all municipal level units in Korea as a random effect in our analysis to account for overdispersion^22^. Using the GLMM, we also modelled the association between breast cancer participation rates and a continuous variable—the original form of the deprivation index. Statistical significance was set at p-value < 0.05. All statistical analyses were performed using Statistical Analysis Software (SAS) (version 9.4; SAS Institute Inc., Cary, NC, USA).

### Ethics approval

The study was conducted in accordance with the Declaration of Helsinki, and approved by the National Cancer Center Institutional Review Board of Korea (approval number: NCC2021-0264, approval date: 2022.08.24).

## Results

Table 1 shows the number and percentage of participants screened for breast cancer by age in 2005, 2012, and 2018. Among all age groups, participants in the 60–69 years age group consistently had one of the highest participation rates in 2005, 2012, and 2018 (2005: 30.37%, 2012: 61.57%, 2018: 65.88%). Meanwhile, participants ≥ 80 years had the lowest screening participation rate in all years (2005, 4.56%; 2012, 16.40%; 2018, 18.78%).

**Table 1:** National breast cancer screening participation rates by age in 2005, 2012, and 2018.

Variable	2005		2012		2018	
	Number of participants screened	Screening participation rate	Number of participants screened	Screening participation rate	Number of participants screened	Screening participation rate
Age						
40–49	264,178	23.40%	534,426	45.70%	611,508	56.33%
50–59	242,206	32.65%	638,195	55.50%	797,091	60.76%
60–69	150,080	30.37%	369,281	61.57%	571,308	65.88%
70–79	61,391	17.45%	194,291	46.63%	231,620	52.54%
≥ 80	7,978	4.56%	37,538	16.40%	58,609	18.78%

The GLMM analysis results of the association between deprivation index quintiles and age-adjusted breast cancer screening participation rates, with municipal level variable added as a random effect, are presented in Table 2. As the deprivation level increased, the breast cancer screening participation rate also increased (2nd quintile; estimate: 1.044, p = 0.242, 3rd quintile; estimate: 1.153, p = 0.192, 4th quintile; estimate: 3.517, p = 0.001, 5th quintile; estimate: 6.913, p =  < 0.0001), when compared with the least deprived quintile (1st quintile) in 2005. In 2012, the participation rate also increased as the level of deprivation increased (ref. 1st quintile; 2nd quintile, estimate: 0.693, p = 0.327; 3rd quintile, estimate: 0.675, p = 0.036; 4th quintile, estimate: 0.760, p = 0.024; 5th quintile, estimate: 0.774, p = 0.0002). There were no statistically meaningful results in 2018.

**Table 2:** GLMM analysis results of Deprivation Index and Breast Cancer Screening Participation Rates in 2005, 2012, and 2018.

Deprivation index	Age-Standardized breast cancer screening participation rate								
	2005			2012			2018		
	B	S.E	P-Value	B	S.E	P-Value	B	S.E	P-Value
1 (Least Deprived)	Ref			Ref			Ref		
2	1.044	0.857	0.242	0.693	0.692	0.327	0.731	0.679	0.290
3	1.153	0.844	0.192	1.508	0.675	0.036	− 0.195	0.674	0.774
4	3.517	0.900	0.001	1.844	0.760	0.024	− 0.227	0.732	0.758
5 (Most Deprived)	6.913	0.910	< 0.0001	3.489	0.774	0.0002	1.448	0.735	0.059

**Figure 1:**
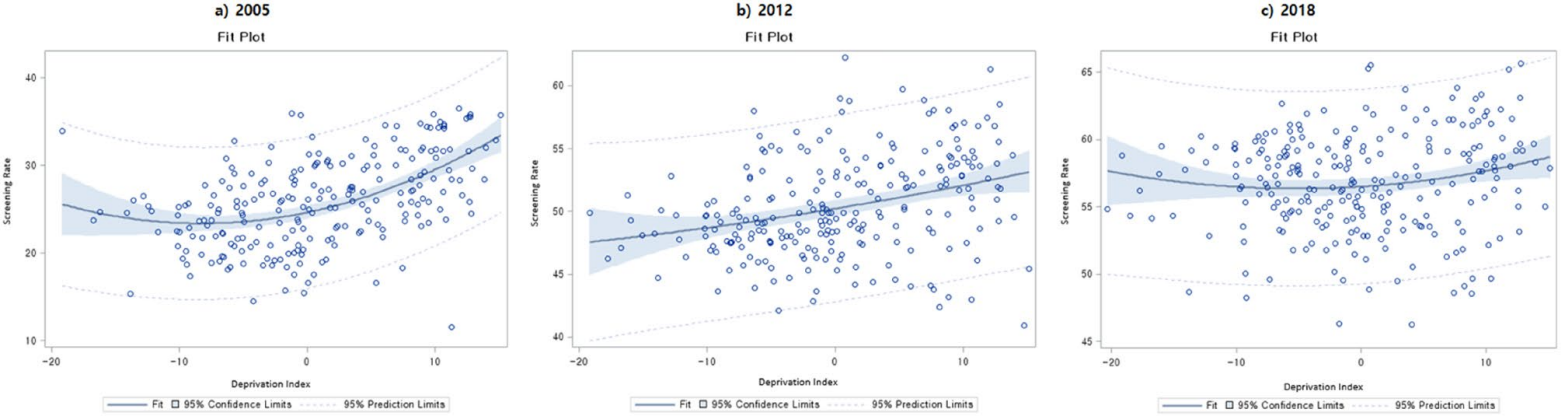
Association between the continuous deprivation index and age-standardized screening participation rates in (**a**) 2005, (**b**) 2012, and (**c**) 2018.

Figure 1 illustrates the GLMM analysis results for the original continuous deprivation index against age-standardised participation rates. In 2005 and 2012, it was shown that as the level of deprivation increased, the participation rate in breast cancer screening also gradually increased. However, in 2018, a slight U-shaped curve was shown when plotted in figure form, indicating that breast cancer screening participation rates were relatively higher at both ends of the deprivation index.

## Discussion

The results of our study suggest that there are no clear disparities in national breast cancer screening participation rates among women in South Korea. Deprivation quintiles are commonly used in health disparity research, but have the disadvantage of only capturing the extreme ends of deprivation. Therefore, we additionally used the deprivation index in its original form as a continuous variable to observe the influence of the full range of the deprivation index on breast cancer screening rates.

Interestingly, areas with greater deprivation generally had higher breast cancer screening participation rates than those in less deprived regions. Given that the NCSP is a population-based organised screening program that offers screening services almost free of charge, it is possible that more socioeconomically disadvantaged individuals are encouraged to make use of these subsidized services. One study by Kim et al. stated that the introduction of the NCSP helped reduce disparities in breast cancer screening in Korea by lowering out-of-pocket expenses^23^. On the other hand, individuals in less deprived areas were less prone to participate in national cancer screening. We speculated that women living in more affluent regions in Korea would seek private opportunistic breast cancer screening programs rather than organised screening, as their high socioeconomic position allows them to opt for the more expensive opportunistic screening, with convenience being one of the considerations behind this choice^24^.

This is also in accordance with a previous Korean study that explored socioeconomic disparities in organised versus opportunistic screening and showed that women in the lower income quartile were more likely to participate in organised cancer screening rather than opting for opportunistic screening^25^. Another study using data from the Korean National Cancer Screening Survey found significant household income inequalities in both cervical and breast cancer screening among socioeconomically disadvantaged Korean women^22^. Education-related disparities were less pronounced but nevertheless significant^26^.

Similarly, a study conducted in France investigated the influence of deprivation on the rate of participation in the French National Breast Cancer Screening Program (NBSCP)^14^. The authors concluded that deprivation had little impact on geographical variations in breast cancer participation rates, and speculated that opportunistic screening may be a key factor in addressing and understanding these variations^17^.

On the contrary, a cross-sectional study carried out in the US Midwest population demonstrated that area-level deprivation and rurality were associated with lower rates of cancer screening participation, including breast cancer screening^23^. The study concluded that rurality and deprivation measures should be accounted for simultaneously when improving screening practices^27^.

Furthermore, a European systematic review reported that women living in more deprived areas were less likely to attend breast cancer screening programs, while the opposite was true for socioeconomically advantaged women^24^. The authors speculated that this phenomenon may be linked to the ‘fundamental cause theory’ where those with higher socioeconomic status had greater access to resources and state-of-the art medical technology^28^. In addition, prior research has reported that individuals with high socioeconomic status were more prone to health-seeking behaviours, including attending regular cancer screenings^29,30^. It appears that socioeconomic inequalities in cancer screening are more pronounced in countries that do not provide population based screening, further highlighting the need for the establishment of high-quality universal screening programs to mitigate disparities across the socioeconomic gradient^31^.

Overall, breast cancer screening rates among Korean women of all ages increased exponentially, as seen from the large jump in participation rates in 2012 and 2018, compared to the early stages of the screening program in 2005. This may be due to increased awareness of the importance of screening in early detection and prevention of cancer^32^. Over the years, the NCSP has put a constant effort to promote cancer screening through the use of TV advertisements and social media campaigns. In addition, invitations in the form of text messages are sent out several times a year to eligible individuals in order to remind them to attend cancer screening. There has been no change in this approach and no new incentives given out by the National Cancer Center. Still, these aforementioned approaches and the public’s overall increased interest in health may have played a crucial role in steadily increasing cancer screening participation rates. Despite this, we found that breast cancer participation rates were noticeably lower in women aged ≥ 80 years than those in women aged 40–79 years. There is no upper age limit in NCSP breast cancer screening, and all eligible women in Korea aged ≥ 40 years are invited to participate in national breast cancer screening^33^.The low rate of participation in older women may be explained by the hesitancy to follow up on screening recommendations. Additionally, concerns that services offered by the NCSP may be of poor quality compared to private opportunistic screening may be another reason for lower screening participation rates^34^. Although there has been a significant improvement in breast cancer screening participation rates across all age groups over the years, efforts to increase the national average breast cancer screening rates in the Korean population are warranted.

A few limitations can be recognised in this study. First, important variables that may have influenced screening behaviour, such as psychological factors (fear of diagnosis) or family history of cancer were lacking. Second, the screening history was limited to that offered by the NCSP, as we only used the NCSP database. Therefore, we were unable to consider the use of opportunistic screening programs, which may have led to an underestimation of screening participation rates.

Despite these limitations, the main of strength of this study lies in the fact that it is one of the first studies in Korea to examine the presence of health disparities in cancer screening by investigating the relationship between deprivation level and participation in national breast cancer screening rates. Many prior studies have investigated the influence of individual socioeconomic factors, such as income or education, on breast cancer screening, but none have used a composite deprivation score to explore this relationship. Another strength is that we used nationally representative data, as both screening and deprivation information were obtained from regions across Korea.

## Conclusions

Our study results suggest that there are no clear disparities in breast cancer screening participation rates among women of different socioeconomic groups. Moreover, evidence suggests that despite the higher participation of women with a high level of deprivation in the NCSP, health disparities continue to exist in the overall cancer continuum^35^. Therefore, we believe it is necessary to investigate how deprivation perpetuates disparities in other stages of the cancer continuum, such as cancer incidence and mortality, as well as in other cancer types. Policy implications regarding the use the deprivation index to quantify health disparities may include establishment of a system to prioritise allocation of needed health resources to regions with high deprivation^36^. The UK, for example, has developed an approach to use their national deprivation index to distribute health service resources equitably based on differences of health needs in the population. This entailed resource allocation including health service investment to the fifth of the local authorities with the worst deprivation indicators. Consequently, one study analysed the effects of this policy and concluded that a greater reduction in mortality amenable to healthcare was found when compared to allocating those resources to more affluent areas^37^. Thus, targeted interventions and health policies are required to identify solutions to improve cancer outcomes and patient centered care, particularly among socioeconomically deprived strata.

## Data Availability

Data is available upon reasonable request to the corresponding author.
